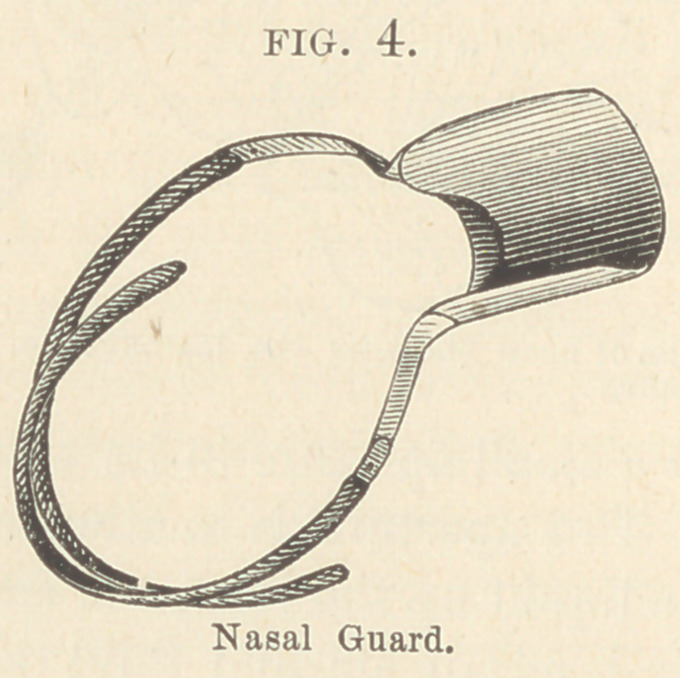# Removal of Hardened Secretions from the Nasal Passages

**Published:** 1877-08

**Authors:** Thos. F. Rumbold

**Affiliations:** St. Louis, Mo.


### EXAMINER.

Vol. XXXV.—AUGUST, 1877.—No. 2.

### Original Communications.

### REMOVAL OF HARDENED SECRETIONS FROM THE NASAL PASSAGES.

By Thos. F. Rumbold, M. D., St. Louis, Mo.

For those patients in whom the muco-purulent secretions have become so hardened, and adhere so tenaciously to the mucous membranes of the superior portions of the nasal and pharyngo-nasal cavities, that their removal cannot be accomplished by force of water inhaled from the palm of the hand, such other means must be resorted to as possess the required force.

There are three indications that must be fulfilled by the means employed to accomplish the removal of these hardened secretions, and cleansing the surface covered by them.

The first of these indications, is, that of itself, the means should cause no irritation.

All who have had even a few years experience in the treatment of this most tenacious disease, will see the necessity of making this indication a prominent one; one that is to measure not only the value of the means for making applications to these highly sensitive surfaces, but to measure the value of the medicaments also that are to be applied to them. It must be constantly kept in mind, that increase of irritation and decrease of chronic catarrhal inflammation can never go on together.

The second indication: The means employed should throw the irrigating fluid upon every portion of the diseased surfaces.

The third: Force enough should be employed to remove the secretions from their places of lodgment.

It is almost needless to say, that whatever means may have been recommended, which will not fulfill these three important and indispensable indications should be discarded.

In discussing the value of the means employed, I will take such only of them as have been recommended by high authority during the last few years.

The Posterior Nares Syringe has been recommended and employed for this purpose, but even when patients have learned to handle this instrument carefully, it so frequently causes, by its application behind the soft palate, so much irritation, that they soon refuse to use it. Besides this, the throat of those patients whose nasal cavities most require to be cleansed by it, are always exceedingly sensitive to all such appliances, and because of the elevation and compression of the velum palati to the posterior wall of the pharynx, occasioned by this sensitiveness, the curved extremity of the instrument is pressed—in the endeavor to insinuate it behind the velum—against this wall with so much force that it soon occasions a pharyngitis even if none had existed before its applications, thus, not only maintaining, but increasing any inflammation that may exist in this region. Not unfrequently, in cases of severe pharyngitis, the application of the instrument is followed by a show of blood in the expectorations, which may continue for fifteen or twenty minutes afterwards. It is preposterous to expect that a naso-pharyngitis can be eradicated under such circumstances. It will require at least two weeks careful treatment to overcome the injury done by one such application.

One of the strongest advocates of this method of cleansing the nasal cavities, a specialist and a resident of an Eastern city, applied this instrument daily for three weeks to a patient, who was himself a physician, each application caused a hemorrhage from the pharyngo-nasal cavity. At first the hemorrages were slight, but they continued to increase, until at the end of three weeks, as much as a half teaspoonful of blood followed the withdrawal of the instrument, at the same time deglutition was so painful as to necessitate a soft diet. The patient was treated for a nasal catarrh, and a deafness in the left ear, but while the secretions in the nostrils were much lessened in quantity, the hearing was rapidly decreased. The practitioner informed his patient that the hemorrhages occasioned by the application of the instrument were beneficial to the mucous membranes, on account of their congested condition, but said nothing about the manifest injury done to the hearing that was occasioned by bruising the sensitive membranes in the neighborhood of the Eustachian tubes.

On account of the irritation caused by the application of this instrument, its use should be discontinued.

The apparatus that is most commonly resorted to, in such cases, is the Weber Nasal Douche. On account of the frequency of the employment of this means, both by the professional and non-professional, I will discuss its merits and demerits at some length, while examining as to whether it can or cannot fill the three indications that I have named.

Dr. Thudichum in his paper published in the London *Lancet* 1864, says: "All difficulties are removed at one stroke by the discovery of Prof. Weber, of Halle, [Germany,] that when one side of the nasal cavity is entirely filled through one nostril with fluid by hydrostatic pressure, while the patient is breathing through the mouth, the soft palate completely closes the channel, and does not permit any fluid to pass into the pharynx, while the fluid easily passes into the other cavity, mostly around and over the posterior edge of the septum narium, in some persons also the frontal sinuses, and escapes from the other open nostril, *after having touched every part of the first half of the cavity of the nose*,* and a great part certainly of the lower and median canal of the second half. By means of the

**The italics are mine. R.*

application of this principle to the treatment of diseases of the nose, it is possible easily and frequently to wash the nasal cavity, to disinfect and deodorize it, to remove the sordes which accumulate so easily in it, and to apply to its surface a great number of beneficial medicinal substances, so as to prevent acute affections from extending, and to incline them towards a speedy recovery; to stop hemorrhages, allay irritations, and subdue in a remarkable manner chronic affections of the Schneiderian membrane, *so as to re-establish perfectly healthy surface and normal condition of the organ of smell*."*

There is an uncommon amount of confidence expressed in the very forcible language just given. It is this confident tone, in which a seeming guarantee of a cure is given, that raised high the hopes of both the practitioner and the patient. It is not questioned but that this douche fulfills the first indication named, *i. e.*, it causes no irritation by its application. This is one of the good qualities that its friends have urged in its favor; it is not uncommon for them to apply to it the adage, which is so frequently applied to homeopathic remedies,namely; that if it can do no good, surely, by its simple action, it can do no harm. Whether the latter conclusion is true or not we shall be better able to see as we go along.

That Dr. Thudichum made a very great mistake when he said that the irrigating fluid touched every part of the nasal cavity—the second indication—may be proved most conclusively by the following experiment: First, cover the mucous membranes of both nasal cavities of the person upon whom the experiment is to be tried, with finely powdered starch, by insufflation, both in front and from behind the velum palati; next, incline the head forward, as recommended by Thudichum, and pass a weak solution of iodine and iodide of potassium through the nasal passages by means of the douche. The iodine solution will either discolor or wash away all of the starch within its reach; the discoloration will be the characteristic blue of iodide of starch. The effect of the washing may be seen by reflecting natural light upon a pharyngeal mirror, placed under and behind the pendant soft palate, and by inspection through the anterior nares. The washed or dis-

*The italics are mine. R.

colored portion of the mucous membrane, and the remaining portion covered with white starch, will show that the greatest height that the iodine solution reached in the antero-superior portion of the cavity, was only a little above the anterior extremity of the middle turbinated process, (b. Fig. 1.) and that

FIG 1.

Antero-posterior section of the head and face, showing the turbinated processes *a*, *b*, *c*; *d*, *d*, the location of the encrusted secretions in the highest portion of the nasal cavity; *e*, the highest the water attains in the nasal cavity while using the Weber douche, with the head inclined forward. The dotted line indicates the position of the posterior border of the septum nasi.

only below a line drawn from this point to the lower surface of the posterior nasal opening (e) is washed, and that all of that portion of the surface above and posterior to that line (d, d,) is not washed, the white powdered starch remaining plainly in view. In other words; the solution, flowing into the nasal cavity, rises until it reaches a level that is on a horizontal line (e) with the inferior surface of the posterior nasal opening of the side in which the liquid is introduced, then, instead of rising higher, upon the introduction of more fluid, it will flow around the posterior border of the septum narium, over that portion of the soft palate which joins the hard palate into the other nasal opening, and thence out through that nasal passage.

Thus, it is seen, that instead of fulfilling the second indication, *i. e.*, " touching every part" of this cavity, as asserted by Thudichum, but a little more than the lower half of it is touched, and it is that half, too, which is very rarely incrusted or requiring treatment; the upper half, the region whence all of the secretions flow that find lodgment in the inferior portion of the passage, remains untouched, and hence uncleansed.

In the other nasal passage, the floor only, not the middle meatus also, as Dr. Thudichum has made us believe, will be washed by the liquid as it escapes.

It is a mistake to suppose that the elevation of the soft palate against the posterior wall of the pharynx will cause the fluid to rise higher in the nasal cavity than the line indicated, because the liquid has still the same avenue for its escape, namely, through the other posterior nasal opening; nor is the closure of the communication downward into the pharynx, a provision by nature,as asserted by the advocates of this method, to allow a more rapid flow of the current into the cavity; nor, indeed, can the nasal fossæ be filled by the closure of the other nostril, because the effect of both of these acts will be to cause the liquid to rise higher, but before the cavity is filled a part of the fluid will flow upon the soft palate, and its presence on this sensitive organ will occasion involuntary deglutition, which will be instantly followed by a sense of strangulation, because the liquid which gave rise to this sensation is not swallowed, but falls down into the open larynx, then a choking sensation of a severe character follows. Even if more of the surface is touched by this forced irrigation, the time during which the liquid is in contact with the higher portion of the cavity is so short that it cannot be effective.

Now, where are the hopes that Dr. Thudichum raised, when he said, that by this means it is possible "to re-establish a perfectly healthy surface, and a normal condition of the organ of smell."

It will appear manifest to all who have studied the anatomy of this portion of the head, that it is not the elevation of the soft palate, nor the closure of the passage into the fauces, nor the closing of both nostrils, but the position of the head of the patient that governs the amount of surface touched by the water. The nasal cavity, while the head is erect, will not retain a liquid any better than a tea cup while lying on its side, but the more that the head is inclined forward, until the posterior border of the septum nasi (dotted curved line) is placed in a horizontal position, the greater will be the quantity of fluid contained in the cavity. But should the douche be employed while the head is in this position, a far more serious inflammation will be set up in other cavities of the head than the one that is being treated, for a part of the irrigating liquid will pass into the antrum of Highmore and a part of it into the frontal sinus, through openings under the middle and superior turbinated processes.

Even if the irrigating fluid did touch every part of the nasal cavity, still it does not fill the third indication, for the stream, as it *must* flow gently, or else involuntary deglutition will take place, does not have force enough to remove hardened secretions, as they are remarkable for the firmness with which they adhere to the place of formation, *i. e.*, in the neighborhood of the superior and middle turbinated processes. Even if two or three gallons of fluid were to be employed—which quantity I have used on several occasions—the time occupied in its passage is not long enough to soften and remove them, yet, by the time that this quantity of liquid ran through the passages, of my patients, the healthy mucous membranes absorbed so much of it as to cause occlusion of the passages to such a degree that they were compelled to breathe through the mouth. After several such applications, such a degree of tenderness was produced, that the least exposure to a cold atmosphere brought on an attack of acute catarrh of portions of the cavity heretofore unaffected.

Although I believe that I have plainly demonstrated that the Weber Douche is inefficient, and might consider that this is a sufficient reason for discontinuing its use, yet I will show that in addition to its inefficiency, it has an injurious effect upon every patient that employs it, by its insidiously spreading the chronic inflammation upon unaffected parts, and that upon some its injurious efforts manifest themselves suddenly and severely.

Before instancing the cases in which the injuries were sudden and severe in character, I must say that the number of persons thus affected, is remarkably small in proportion to the large number who have used and are daily using this method.

There certainly is a very *large* number of persons who are employing this means for cleaning their nasal passages; as fast as one set discontinue its use, after finding out that it does not fulfill their expectations, another set commence it, and yet the cases of acute inflammation of the cavities connected with the nasal passages, that arise from it, *are not* at all frequent. I am now treating a patient who commenced to use this douche in March, 1871, washing his nostrils by it from one to three times, and sometimes as high as four and five times daily; he very rarely passed a day without using it, making in all, certainly, about three thousand applications. Two times during this period, he experienced painful sensations in his ears; four or five times he experienced a painful sensation in his left cheek, showing that the left antrum of Highmore was injuriously affected by it.

It is seldom that I treat a catarrhal patient who has not, in his endeavor to rid himself of this disease, used this douche a great many times, yet it is seldom that complaint is entered against it, on account of any injury received from it, that is, one that the patient or his physician would call an injury; I mean such an injury that would develop itself suddenly, or show itself by symptoms of a marked character; so small, indeed, is the number of cases whose ears and sinuses receive injury of this character, that, in my opinion, were the method as effective as claimed by Dr. Thudichum, it should not be discontinued on account of its effect upon these cases.

It is not because that this method, now and then lights up an acute inflammation in comparatively few cases out of the thousands who use it daily, almost without instruction or warning, that I would condemn it, but it is, on account of the injury that the water does to the healthy surfaces without, at the same time, benefiting the unhealthy or catarrhal surfaces.

The application of water or of any fluid, except mucus, to the nasal cavities, is always productive of more or less injury to its healthy mucous membranes, but this injury is more than compensated, if, by the application, vitiated and irritating secretions are removed, which could not have been done without its aid; but if these secretions are not removed during its application, then the injury done by the water to the healthy parts is not compensated for by any benefit done to the inflamed parts, but on the contrary, the condition of the patient is gradually, almost imperceptibly, made worse by the healthy mucous membrane being prepared, by the frequent absorption of water, so that it more readily takes on a catarrhal inflammation. This is the injury that should deter every one from employing this means. I am quite certain that fully ninety-five per cent. of my patients, who have used this douche, have not only maintained their catarrh by it, but by it caused the chronic inflammation to extend to other parts of the cavity, as well as to other cavities.

It will be seen that what I have said about its liability to set up a chronic inflammation in other cavities, is almost in accordance with Dr. Roosa's experience, given in his work on the ear. He says: "As early as 1869, I had found that the nasal douche was sometimes a troublesome and dangerous appliance, and I added a note to indicate this in my translation of Von Troeltsch on the ear, [second edition, page 369,] but I was not fully convinced that it would readily cause acute aural inflammation until the following case occurred in my practice. * * * Besides, as it is believed by many otologists, it is possible that the douche sets up a chronic inflammation of the tympanic cavity, without any acute stage, and thus the true cause of an insidious chronic catarrh is passed over and supposed to be an advance of the naso-pharyngeal inflammation. Of course it is not believed by the author that the use of the nasal douche will *necessarily* cause aural disease, but that it is a dangerous means of treatment, which should be carefully watched by the practitioner."*

Although Dr. L. Turnbull is a strong advocate of this method, yet it is evident that the facts which he records in his work on the ear are also in agreement with what I have said. He says: "There are some important cautions to be observed: first, the fluid must be of the temperature of the body, [about 96°]; second, the patient must breathe gently with the mouth

**Dr. St. John Roosa on the Ear*, 1873, *pp.* 291-295.

open; and lastly, must not swallow, else the fluid will pass into the middle ear and cause the following results, well told by a patient in the following letter from Frederica, Delaware:

'My Dear Sir:—I find on using the nasal douche as recommended by you, that it affects me somewhat unpleasantly. I find no difficulty in passing the water as directed from one nostril to the other, or back into the throat. On passing the water into the throat the Eustachian tubes apparently are also filled, and give the same sensation I have experienced, when a boy, in swimming, and what we used to call "bubbles in the ear." I cannot free my head of the water taken in for some four or five hours after using the douche. I then feel as if I had taken cold. My ears feel sore, pressing the tips of the fingers into the external ear causes a dull pain, apparently about the drum of the ear. This passes off in about twelve hours. . . . I am much more deaf than usual for some hours after using the douche. Yours respectfully,

J. R. H.'

"To this form of medication there are some other objections which have been made by Professors Roosa and Knapp; viz., that otitis media may supervene, and perforation of the membrana tympani be caused by excessive sneezing, the result of using the douche; but no such results have followed the extensive use of this most valuable means employed by the author in hundreds of cases, both of ear diseases and of ozæna with or without deafness."*

Instead of its being a most valuable means, as claimed by Dr. L. Turnbull, the experiment with the powdered starch and iodine solution demonstrates that it is really valueless, except so far that it makes it possible for those patients who suffer from profuse catarrh to breathe with some degree of comfort, by its removing the secretions that occlude the inferior portions of their nasal passages. It is because of this relief that patients express themselves as pleased with the method. Besides this, the effect of the warm fluid is always pleasant to those patients, even if the whole of the diseased surfaces are

***A Clinical Manual of the Diseases of the Ear*, by *Laurance Turnbull, M. D.*, 1872.

not bathed by it. I have noticed for years that the expressions of benefit or relief almost invariably come from those patients whose nasal cavities were plugged by inspissated secretions, and who suffer in consequence of the heat arising from the inflammation, and not from those whose catarrhal complaint allows a free passage for breathing, except at such times as they suffer from an unusual amount of irritation occasioned by a recent cold.

It is very common for physicians, in reporting the favorable result of the application of this douche in a very bad case, to say, as Dr. Thudichum said: "It is really surprising what an amount of sordes will sometimes be removed from the nose by this rinsing process," or "that great masses of hardened, offensive secretions are washed out, and that this relieved the patient of an ever present weight in the head." Such expressions as these lead the reader of the report, as it led me, to presume that if this method of treatment will produce so marked, so beneficial a result upon so bad a case, it will certainly cure a case that is but slightly affected. But the fact is, so far as relief is concerned, the very reverse of this is true; the cases of profuse catarrh are relieved, but not cured, and the slight cases are injured by it, without experiencing any relief.

That this method will remove the secretions that are situated in the inferior and anterior portions of the cavity, (a. b. Fig. 1.) is not doubted, but this is all that it will do, its usefulness ends here. This removal has the effect to give the patient breathing room only, the disease is not even checked. The larger half of the treatment that is to cure the case, is to remove, completely,the secretions from every portion of the cavity, this, the douche cannot do. The portions of the cavity that are the most important to be cleansed, are the superior portions, (d. d. Fig.1) because the disease originates in this locality, consequently there always is secretion on these surfaces. There are many cases, severe ones too, in whom the lower portion of the passages is entirely clean and healthy, which requires no applications of water, but will be injured by the absorption of the water, if it is applied every day.

I will now relate a part of my experience in the employment of this douche, that I may be enabled to give the history of the circumstances by which I discovered the inadequacy of its applications. Following this, I will give brief histories of those cases who were injured by its applications.

In January, 1863, while located in the U. S. Gen. Hospital, at Jefferson Barracks, Mo., I had two patients under my care who were suffering from nasal catarrh. I directed them to wash out their nasal passages with various solutions, by means of Matison's soft rubber syringe. Other soldiers, noticing the applications, requested to be treated for a similar complaint. During this year and the following one, I treated, or attempted to treat, in all, sixty-eight patients.

The failure to do more than maintain a passage through the nostrils, added to failures that occurred years before, on several cases similarly affected, induced me, in January, 1865, to open a correspondence with a class-mate in Boston, who had recently visited the hospitals in London and Paris. From him I learned of Dr. J. L. W. Thudichum's article on a "New Mode of Treating Diseases of the Cavities of the Nose," which appeared in the London *Lancet*, of November and December, 1864.

These articles contained a full description of the Weber Nasal Douche, and gave a list of remedies to be used. Their tone was so confident and so assuring, that I was ready to conclude, with my friend, that at last we had the means of combating this complaint, which had heretofore baffled all endeavors. At the time of the reception of the two numbers of the *Lancet*, I had six cases of nasal catarrh in my ward, so certain was I of curing them by this method, that I wished that I had sixty cases instead of six.

The patients, at first, were much pleased with the effects of the washing, and I could see that the prominent symptoms were much abated.

In a few weeks, I noticed that it was those patients only, whose nostrils were very much filled by sordes during the night that continued to give the most favorable reports. About four months after I commenced to use this mode of treatment, one patient, on whom the douche had been used about three weeks, refused to have it applied because, as he claimed, it caused intense pain in the left side of his face, in the upper jaw and also in his forehead. Soon after this, another patient informed me that it had the same effect on him, and, moreover, that the secretions from his nose and throat were more profuse than at any time during his life, his catarrh being but a slight one when I commenced to douche him. The first patient that was injured by the washing had an inflammation of the antrum of Highmore on the left side, he insisted that the douche caused it, but I did not think so at the time, because, on examination of his teeth, I found that the second upper molar, whose fang sometimes penetrates into the antrum, was decayed. I extracted this tooth and treated the diseased antrum through the opening made by the tooth. The case, so far as the diseased sinus was concerned, recovered in about five weeks.

As I considered that the decayed tooth originated imflammation of the antrum, I recommended that the patient should use the douche again. He did so, and had four applications, when a very severe inflammation of the antrum again ensued, from this he recovered after about two months close attention. The second case in whom the antrum was involved, did not require any special treatment; I merely let him alone; and in a few weeks he too, recovered, his catarrhal symptoms also improved slightly upon non-interference.

I discovered about this time that while the douche had a good effect on those patients whose catarrh was very profuse, it proved an injury to all cases in whom the secretions were always in a fluid condition and were but small in quantity.

In order to ascertain the reason of this peculiarity, I made an examination, post mortem, of a patient who had died suddenly of a paralysis: he had a very profuse catarrh, and had been treated by the douche about three months. The applications were made daily for ten days, then at such times as the secretions demanded removal, which was about every other day. The treatments gave so much relief when first employed, that he expressed himself as being quite certain that they would ultimately cure him. He had frequently stated that he had never used anything that had so good an effect on him as the douche of warm salt water. I was astonished to find, during the post mortem examination, that the posterior portion of the superior half of the nasal cavities (d, d, Fig. 1) were incrusted with old and offensive secretions, although the passages had been washed out, in accordauce with his request, about six hours before he died, and that he had been regularly douched from the comencement of the treatment.

Having made an antero-posterior section of the head, I made a large opening in the septum nasi and placed over this perforation, a piece of window glass large enough to close the hole, then I inclined the head forward, as recommended by Thudichum, inserted the rubber tube into the nostril and caused water to flow into the cavity, in the same manner that I had done in the treatment of my cases. Through the glass septum, I saw that the water was maintained in the cavity at that height only that was on a level (e, Fig. 1) with the lower border of the posterior nasal opening of the side douched, therefore the irrigating fluid could not wash the superior and posterior portions of the nasal and pharyngo-nasal cavities (d, d, Fig 1); it could wash the inferior and anterior portions only a, b, Fig. 1). This experiment at once solved the mystery of this form of douche being beneficial in cases of profuse catarrh, but never checking entirely the formation of the purulent secretions either in severe cases, or in mild ones. I had then used the Weber Douche eight months, (Sept. 1865 making from five to twenty applications of it every day, and was satisfied that it had gained its reputation from the relief that it had afforded to patients who were suffering with profuse secretions and large incrustations.

As the medical journals continued to praise this method, and as it was the best means known for alleviating bad cases of this disease, I continued its employment until June, 1866, at which time I had two patients (then in private practice) whom I injured by its use. One of them suffered so severely from otitis media that I perforated the membrana tympani; the other had an inflammation of the antrum of Highmore. At this time, partly at the suggestion of a patient, I began to recommend, instead of the douche, the inhalation of water from the palm of the hand, while the head was inclined forward, as recommended in a previous article.*

In September, 1868, I drew the attention of the members of the St. Louis Med. Society to the deficiencies of this douche, by drawings on the blackboard, demonstrating the manner in which the irrigating fluid failed to reach the superior portion of the nasal cavity, and at the same time mentioned two cases which I had treated that year, whose ears were injured by means of this apparatus. In both of those cases a perforation of the membrana tympani had taken place; one of the patients was seriously ill for a period of four weeks from an inflammation of the mucous membrane of the mastoid cells.

In 1869, I treated two cases whose ears were affected injuriously by the douche, on one of whom the mastoid process was greatly swollen, which was relieved by a free incision.

In 1870, I had five cases who were injured by this apparatus. I took the pains to inquire whether or not they had informed the physician who had recommended the douche, of the bad effects of the treatment, and learn from them that they had not done so.

In 1871, I had only one case that was injured by the douche. He had been using the apparatus for about four years, and had repeatedly experienced sensations as if the water had passed in both ears. He had noticed that the solution passed into the ears at such times as he had a cold in the head. He informed me that he knew of several of his acquaintances who were affected in the same way, " but," he said, " each of us had ear-ache when we were young, and I thought that the ear-ache had made our ears weak."

In 1872, I treated four cases from injuries done by the douche. Three of those cases were but slightly affected in the ears; two of the three had otorrhea when young, the third one had no affection of the ear except from the use of the douche. The fourth case was affected in the left antrum of Highmore, a molar tooth, which was partially decayed, was extracted to afford an opportunity to treat the cavity.

**Chicago Med. Jour. and Examiner, vol.* 34, *page* 385.

In 1873, I had two cases, both of whom had otitis media, but neither very severe. No history of previous complaint of otorrhea, but both were quite defective in their hearing before using the douche.

In 1874, I had six cases who had otitis media from the use of the douche, and two cases in which the antra were affected injuriously by this apparatus. All of the cases were mild ones.

In 1875, I had three cases of otitis media from the use of the douche; in one of these cases, which was severe, there is a history of a previous affection of the ear. The hearing of all of the cases was quite defective.

In 1876, I had seven cases of otitis media, and one of inflammation of the antrum of Highmore,and one of inflammation of the frontal sinus. The hearing of the seven ear cases was defective before the use of the douche, but much more so after it had caused inflammation of the middle ear. In two cases I perforated the membrana tympani; in one of those the perforation closed in four days, in the other in about three months. In the case of the inflamed antrum, I had a second molar, which was decayed, extracted to allow the escape of the pus. The case with the inflamed frontal sinus was very severe, the lower portion of the forehead was greatly swollen, and very red. The pain was so great as to prevent sleep for three days.

I have noticed a fact, connected with the history of nearly every one of my cases, which to a certain extent mitigates the blame that is attached to this method, that is, to its exciting acute inflammation in distant parts. The fact alluded to is, that their ears and antra were in a more or less inflamed condition before the application of the douche. In all ear cases, even if there had been evidences of a diseased condition—except in those who suffered from perforation of the membrana tympani—if they desisted from performing the act of deglutition, thus preventing the entrance of water into the middle ear, the employment of the douche did not produce acute inflammation. The ears of those patients whose membrana tympana were perforated, were unaffected by the douche, even if the act of swallowing was performed while the water was in the pharyngo-nasal cavity. I think that it is barely possible for water to enter a middle ear, if its membrana tympani is perforated. I have not seen nor heard of a case in which it did do so. I have also noticed that those patients whose ears had not manifested any symptoms of a diseased condition previous to the use of the douche, did not volunteer complaints of its bad effects, even when the water did enter their ears. But, from my observations, I should expect that in *every* patient whose ears were affected by an *acute* inflammation—except in those in whom the membrana tympani were perforated—

FIG. 2.

Catheter Nasal Douche: *a*, container; *b*, metal tube for passage of the liquid, the *letter* is placed beside a small aperture in the side of this tube, allows the entrance, of air; *c*, hose composed of rubber and glass tubing; *d*, catheter with foramina, for the escape of air and liquid; *e*, triangular piece of soft rubber; *f*, India rubber air bulbs used to force air into the container *a*.

all of the acute symptoms would be suddenly aggravated, if they performed the act of deglutition while employing the douche.

Even if it were possible to determine those patients who should not use this method of cleansing the nasal passages, this fact ought not to be urged against its employment, if it had a salutary effect on all of those cases whose ears and antra were uninjured by it; but when it proves a serious injury to some patients, and when it signally fails, in every patient, to reach the locality in which the disease *originates*, thus returning no compensation for the injury that it must do to *uninflamed membranes, by their absorbing water*, then, most certainly, it should be discontinued.

After observing the inadequacy of the Weber Nasal Douche, I devised an apparatus in June, 1867, which I have called the Catheter Nasal Douche, (Fig. 2). It throws a shower or coarse spray of liquid from the floor of the nostril upward, reaching every portion of the irregular surface of the cavity, making perfectly efficient and direct local application. When warm salt water is used, the only sensation it occasions, is that of tickling, which is never objected to by the patient.

The apparatus consists of the following parts: The vessel that contains the cleansing fluid is a flask-shaped bottle (a, Fig. 2) of a pint or a pint and a half capacity; into the soft rubber stopper of this bottle are inserted two metallic tubes, whose outer extremities are bent at right angles, and turned in opposite directions. One of these tubes is short, but long enough to pass through the stopper, and has attached to its outer extremity a pair of India rubber air bulbs (f); the other metal tube (b) almost reaches the bottom of the container. Attached to the outer extremity of this tube is a hose (c), about twelve inches long, a part of which consists of soft rubber and a part of glass tubing, the latter section of tubing is about three inches long, and is inserted in the first third of the hose. To the outer extremity of the hose is fastened a No. 5, or No. 6, flexible catheter (d) six inches long, at the further end of which are made five small openings in a line with its axis, three-eighths of an inch apart. The free extremity of the catheter is closed. A perforated triangular plate (e) of soft rubber, with one inch borders, is slipped on the catheter about three and a half inches from the closed extremity. This plate will prevent the liquid from flowing on the operator's hand, and at the same time it will serve as a guide both in regard to the direction of the stream and the distance that the instrument is inserted into the nostril (Fig 3).

The metal tube, whose lower extremity dips into the fluid in

FIG. 3.

Antero-posterior section of head showing the introduction of the catheter and the direction of the coarse spray.

the container, has a small aperture in its side, just under the rubber stopper. This aperture is to allow air to enter during the passage of the liquid up the tube, the effect of which is to cause it to contain beads of air and fluid alternately. These beads of air and liquid should be equal in size, about one-half of an inch long. When the air and solution escape from the opening in the catheter (d), it will resemble a coarse spray. The relative size of the beads and water may be ascertained by suddenly arresting the current in its passage through the hose, by compressing the rubber tubing near the catheter, and inspecting at the glass section of the hose. If the air beads are relatively the larger, then the aperture (b) under the rubber stopper, in the long metalic tube, is too large; if the air beads are smaller than the water beads, then the aperture is too small. In either case the aperture should be so made that the beads will be about equal in size.

In its application, the catheter is introduced horizontally into the nasal cavity to be cleansed (Fig. 3). The coarse spray or spattering current of liquid and air is made to pass directly upward; by slight rotation of the instrument on its axis, the stream will wash and blow the secretions from their lodging places under the turbinated processes, and in the highest portion of the cavity, in a much milder manner than a steady stream from any form of a syringe applied either in the anterior or posterior nasal openings, and in a much more efficient manner than the Weber Nasal Douche.

The cleansing process may be greatly assisted by the patient closing the nostril not treated, and then giving a quick and forcible blow out of the one that is being washed, this will expel the liquid and everything loose with considerable force.

A nasal guard, (Fig. 4) fitted on the head so that it may be

FIG. 4.

Nasal Guard.

placed under the nose, will prevent the irrigating solution and the muco-purulent secretion from falling on the lips, and from soiling the clothing at such times as the patient is blowing his nose.

This apparatus, if care is taken not to force too much air into the reservoir, fills all three of the indications that are required to properly cleanse these cavities. 1st, It does not produce irritation; 2d, it throws the irrigating fluid to all parts of the nasal cavity, even under the turbinated processes; and, 3d, it has force enough to remove all of the hardened secretions, and cleanse the surfaces after they are removed. This force is completely under the control of the patient or the person employing the apparatus, so that the coarse spray of air and liquid may be caused to strike the secretions with such force only as is required to remove them, and after the removal, the force may and should be lessened, to complete the cleansing.

The amount of fluid that is employed is a matter of great importance. We must keep in mind that the mucous membranes, especially that portion of them that are in a healthy condition, absorb to their injury more or less of every liquid that comes in contact with them; for this reason the application of the water should be discontinued just so soon as the hardened secretions are removed, even if the washing process produces a pleasing sensation. If the washings are protracted, the healthy mucous membranes in the lower portion of the nasal cavities will absorb so much water, that they will become swollen, in which condition they are more liable to be injured by the influences of out-door atmosphere.

If at any time the force of the stream is such as to produce a painful sensation, which lasts beyond one or two seconds, then the washing should be discontinued, even if the passages are not entirely cleansed. If the disagreeable symptions pass off in a few seconds, the washing might be commenced again, but with such force of the stream as to produce no disagreeable sensation; if the pain occasioned by the first effort, lasts beyond one minute, then the washing should be deferred for several hours.

The washings should be done in the morning before breakfast, and repeated often enough to keep the passages free of hardened secretions, but each time using as *small an amount* of fluid as will accomplish the cleansing process.

As soon as the secretions cease to become hardened, the washing by the Catheter Nasal Douche may be discontinued, and the inhalation of the water from the palm of the hand substituted, as the latter mode is sufficiently effective, and is accomplished with much less trouble.

The irrigating solution is made by dissolving in a pint of water, that is a little warmer than blood heat, about one teaspoonful of common table salt. Patients will soon learn from experience whether or not this is the proper strength and temperature. Water either without salt or with too much in it, produces more or less pain, yet, with the right quantity (which varies slightly with different individuals), it produces a pleasant, bland sensation. Cold water causes a disagreeable as well as an injurious effect.

For those cases in whom the secretions are offensive, five grains of salicylic acid should be added to the pint of solution.

## Figures and Tables

**FIG 1. f1:**
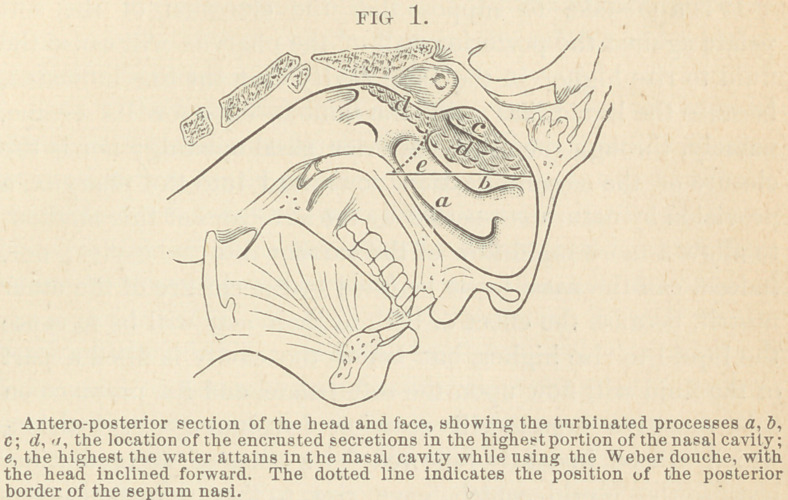


**FIG. 2. f2:**
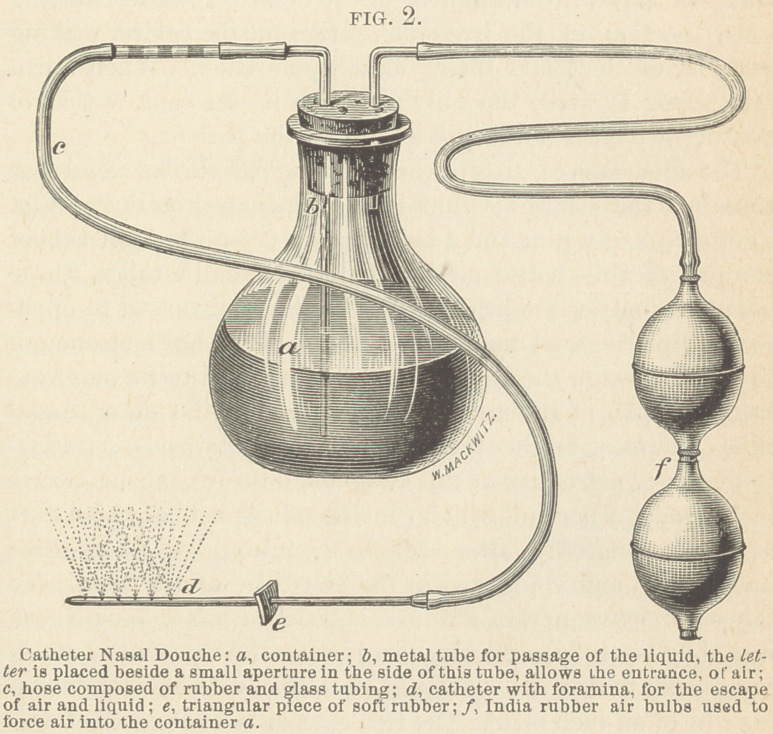


**FIG. 3. f3:**
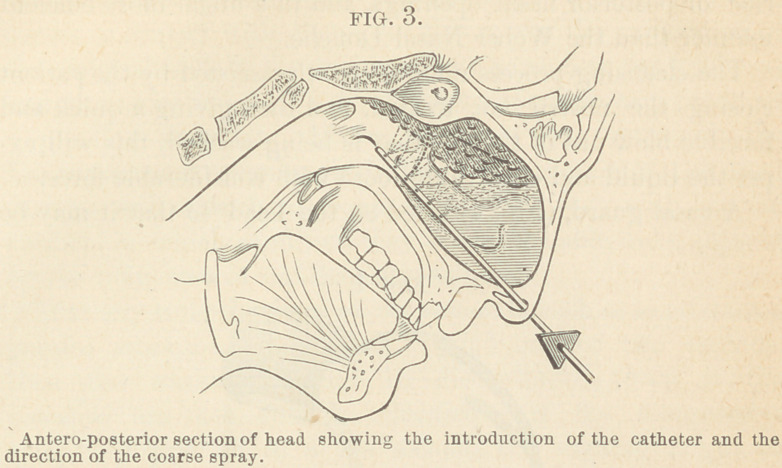


**FIG. 4. f4:**